# Supplementary material for: Patterns of Intron Gain and Loss in Fungi
Source: PLoS Biol. 2004 Nov 30;2(12):e422. doi: 10.1371/journal.pbio.0020422 (PMC532390; doi:10.1371/journal.pbio.0020422)
Supplement: Table S1 — Also available at http://genes.mit.edu/NielsenEtAl/. (4.3 MB ZIP). [file pbio.0020422.st001.zip › NielsenEtAl/html/1221.html]

AN5800.1.NCU03988.1.MG04484.1.FG09866.1


```
 CLUSTAL W (1.82) Multiple Sequence Alignments - Introns Inserted


Sequence 1: NCU03988.1	183 aa
Sequence 2: MG04484.1	184 aa
Sequence 3: AN5800.1	184 aa
Sequence 4: FG09866.1	184 aa
Alignment Length: 184 aa
Number Identitical Residues: 135 aa
Alignment Score (without introns) 5722


MG04484.1 	MG1IDLKKHHVRSTHRKAPKSDNVYLKLLVKLYRFLAR1RTDSSFNKVVLRRLFMSRTNR
NCU03988.1	MG1IDLRKHHERSTHRKAPKSDNVYLKLLVKLYRFLAR1RTDSAFNKVVLRRLFMSRINR
FG09866.1 	MG1IDLDRHHVKGTHRTAPKSDNVYLKLLVKLYRFLAR1RTDASFNKVVLRRLFTSKINR
AN5800.1  	MG1IDLDRHHVRSTHRKAPKSENVYLQVLVKLYRFLAR1RTESNFNKVVLRRLFMSRINR
          	** *** :** :.***.****:****::********** **:: ********** *: **

MG04484.1 	PPVSLSRIAGNLKNGNEKKTVVVVGTVTDDNRLVECPKAQVAALRFTATARARIVAAGGQ
NCU03988.1	PPMSISRIVANLK-GNEKKTVVVVGTVTDDNRLLTVPKVSVAALRFTATARARIEAAGGQ
FG09866.1 	PPVSLSRIVANINKEGEKRTVVVVGTITDDNRLLECPKVTVAALRFTATARARILAAGGE
AN5800.1  	PPVSLSRIASNVTESHQGKTIVVIGTVTDDNRLLTVPKLSVAALRFTATARARIEKAGGE
          	**:*:***..*:..  : :*:**:**:******:  **  **************  ***:

MG04484.1 	AITLDQLALEKPTGANTLLLRGPKNAREAVKHFGFGPHKHK0KPYVQSKGRKFEKARGRR
NCU03988.1	AITLDQLALEKPTGANTLLLRGPKNAREAVKHFGFGPHKHK0KPYVASKGRKFEKARGRR
FG09866.1 	AITLDQLALRAPTGSNTLILRGPKNAREAVKHFGFGPHSHK0KPHVESKGRKFERARGRR
AN5800.1  	TLTLDQLALRAPTGANTLLLRGPKNAREAVKHFGFGPHKHK0KPYVRSKGRKFERARGRR
          	::*******. ***:***:*******************.** **:* *******:*****

MG04484.1 	RSRGFKV
NCU03988.1	RSKGFKV
FG09866.1 	RSRGFKV
AN5800.1  	RSRGFKV
          	**:****
```
